# Use of Eculizumab in Pediatric Patients With Transplant Associated Thrombotic Microangiopathy

**DOI:** 10.3389/fped.2021.761726

**Published:** 2021-11-11

**Authors:** Laura Gomez-Ganda, Maria Isabel Benitez-Carabante, Aurora Fernandez-Polo, Marina Muñoz-Lopez, Berta Renedo-Miro, Gema Ariceta, Cristina Diaz De Heredia

**Affiliations:** ^1^Pharmacy Department, Vall d'Hebron University Hospital, Barcelona, Spain; ^2^Pediatric Oncology and Hematology Service, Hematopoietic Stem Cell Transplantation Section, Vall d'Hebron University Hospital, Barcelona, Spain; ^3^Pediatric Nephrology Department, Vall d'Hebron University Hospital, Barcelona, Spain

**Keywords:** thrombotic microangiopathy (TA-TMA), hrTA-TMA, eculizumab, complement system, hematopoietic stem cell transplant (HSCT), CH50, complement inhibitor, sC5b-9 (serum complement membrane attack complex)

## Abstract

**Background:** Transplant-associated thrombotic microangiopathy (TA-TMA) is a serious complication of hematopoietic stem cell transplantation (HSCT) associated with high morbidity and mortality. High-risk TA-TMA (hrTA-TMA) is characterized by multifactorial endothelial damage caused by environmental stressors, dysregulation of the complement system, and genetic predisposition. Complement inhibitors have significantly decreased mortality and are the current treatment of choice. In this article, we describe our experience with the use of eculizumab in pediatric patients diagnosed with hrT-TMA after HSCT.

**Method:** Retrospective study of pediatric patients with hrTA-TMA treated with eculizumab between January 2016 and December 2020.

**Results:** Four pediatric patients aged 1, 12, 14, and 17 years at the time of HSCT were diagnosed with hrTA-TMA and treated with eculizumab during the study. At diagnosis, they all had renal impairment with proteinuria, and hypertension under treatment with at least two antihypertensive drugs. The patient who presented multisystemic involvement died instead of treatment. The three patients with exclusive renal involvement achieved TA-TMA resolution after treatment with eculizumab for 65, 52, and 40.6 weeks and were able to stop treatment. The two patients with follow-up data one year after eculizumab withdrawal sustained a favorable response. Eculizumab was well tolerated, and with adequate vaccination and antibiotic prophylaxis, did not increase the risk of infection.

**Conclusions:** Eculizumab appears to be both safe and effective for the treatment of hrTA-TMA in patients with renal impairment. Early diagnosis and initiation of treatment may improve response. Eculizumab withdrawal can be contemplated in patients who achieve laboratory and clinical resolution of TA-TMA.

## Introduction

Transplant-associated thrombotic microangiopathy (TA-TMA) is a serious complication of hematopoietic stem cell transplantation (HSCT) associated with high morbidity and mortality. Survival in this setting, however, has improved significantly since the introduction of complement inhibitors (1–4).

TA-TMA is characterized by multifactorial endothelial damage caused by environmental factors (chemotherapy, total body irradiation [TBI], human leukocyte antigen [HLA] mismatch, calcineurin inhibitors [CNIs], graft-vs.-host disease [GVHD], opportunistic viral and fungal infections, and antiviral drugs); complement dysregulation; and, in some cases, genetic predisposition (1, 2, 5–10). Patients with genetic variants and polymorphisms in genes involved in complement regulation seem to have increased susceptibility to TA-TMA (11).

The kidneys are often the main organs affected in TA-TMA, but this multisystem disease can also affect the lungs, heart, gastrointestinal tract, and central nervous system (1–3, 9, 12, 13). Early diagnosis is challenging due to the nonspecific nature of manifestations and the clinical and laboratory characteristics of patients following transplantation. The most widely used diagnostic criteria for high-risk TA-TMA (hrTA-TMA) in children were proposed by Jodele et al. (2) (Table 1).

**Table 1:** Diagnostic criteria for high-risk TA-TMA proposed by Jodele et al. (2).

**Histologic diagnosis**
TMA confirmed by tissue biopsy
**Laboratory and clinical diagnosis (5 of 7 risk markers are required to make TA-TMA diagnosis, but features 6 and 7 should be present for high-risk TA-TMA diagnosis)**
1. LDH elevated above the upper limit of normal for age
2. Presence of schistocytes in peripheral blood
3. *De novo* thrombocytopenia or increased transfusion requirements
4. *De novo* anemia or increased transfusion requirements
5. Hypertension > 99% for age <18 years of age
6. Proteinuria (≥ 30 mg/dL x 2 or random urine protein/creatinine ratio ≥ 2mg/mg)
7. Elevated plasma concentration of sC5b-9 above upper normal laboratory limit
**TA-TMA with multiorgan dysfunction syndrome (MODS)**
5 of 7 high-risk markers present and must include markers 6 and 7
Evidence of MODS
Histologic evidence of TMA on a tissue specimen

*LDH, lactate dehydrogenase; MODS, multiorgan dysfunction syndrome; sC5b-9, soluble terminal complement complex; TA-TMA, transplant-associated thrombotic microangiopathy; TMA, thrombotic microangiopathy*.

TA-TMA treatment should be individualized in accordance with disease severity and clinical status. First-line therapy consists of minimizing exposure to potential triggers. This includes CNI withdrawal or dose reduction or replacement with an alternative immunosuppressant, such as mycophenolate mofetil (MMF). Careful monitoring for GVHD is essential in all cases (1, 2, 4, 6, 8, 13).

Previous treatments for hrTA-TMA included plasmapheresis, rituximab, and defibrotide, but their use was based on case series and retrospective studies and associated with high mortality (2, 5, 6). Evidence supporting a key role for complement dysregulation in TA-TMA led to the off-label use of complement inhibitors as first-line treatment. Eculizumab is a humanized monoclonal antibody against complement protein C5 that prevents tissue damage by blocking formation of the membrane attack complex (C5b-9). Early initiation of treatment is essential to achieve adequate response and hrTA-TMA resolution (1, 7, 9).

Publications from a small number of expert centers recommend individualized pharmacokinetic monitoring to assess plasma levels of eculizumab (1–3, 6, 13). The technology needed, however, is not widely available and results can take time. Serum sC5b-9 (soluble membrane attack complex) and CH50 (total hemolytic complement activity) have been proposed as more accessible and rapidly available complement blockade markers (1, 4, 13). Endothelial risk factors associated with HSCT decrease during follow-up, and the tissue damage responsible for hrTA-TMA also diminishes as normal complement activity is restored. Since hrTA-TMA is a secondary form of TMA, eculizumab withdrawal can be considered in patients who respond favorably (1, 4, 12–14).

Patients' survival has improved significantly with the use of eculizumab in hrTA-TMA, with one pediatric cohort reporting a survival rate of 66% 1 year after HSCT (13). Data, however, are scarce and are mainly based on retrospective observational studies. In this article, we share our experience with the use of eculizumab to treat hrTA-TMA in pediatric patients following HSCT. Although our series is small, we believe that our findings will help guide clinical practice in the treatment of this serious complication.

## Methods

### Study Design

Retrospective study of pediatric patients (aged ≤ 18 years at HSCT) with hrTA-TMA treated with eculizumab between January 2016 and December 2020 at a pediatric university hospital.

Demographic, clinical, and treatment data were obtained from electronic health records and computer-assisted prescription systems. Ethical standards and legal requirements on the use of personal data were applied throughout the data collection stages. The use of eculizumab was approved by the institutional review board and the patients' parents or legal guardians gave their informed consent for its off-label use.

### Study Endpoints

The main objective of our study was to share our experience with the use of eculizumab to treat hrTA-TMA in pediatric patients and to increase awareness of this serious complication. Our specific aims were to (i) define patient, clinical, and laboratory characteristics that could influence response to eculizumab treatment and (ii) study clinical outcomes following withdrawal of eculizumab after hrTA-TMA resolution.

### TA-TMA Screening

All HSCT patients at our hospital undergo strict blood pressure (BP) management and regular monitoring of renal function, blood count, hemolysis markers, and proteinuria. The clinical diagnosis of hrTA-TMA was established according to the criteria proposed by Jodele at al. (2) and confirmed by renal biopsy in stable patients. Cardiac and ocular impairment were evaluated by echocardiography and funduscopy.

Diagnoses of immune hemolytic anemia and thrombotic thrombocytopenic purpura were ruled out by the direct antiglobulin test (DAT) and quantification of ADAMTS-13 activity.

Biochemical, immunological, and genetic studies of the complement system were performed to investigate polymorphisms and risk variants for TA-TMA and rule out primary TMA. Because the patients had undergone HSCT, these studies were conducted using a saliva swab sample.

The biochemical and immunological studies of complement proteins included determination of plasma levels of C3, C4, factor I, and factor H; complement factor H (CFH) activity (functional assay); anti-factor H antibodies; and membrane cofactor protein (MCP) levels in polymorphonuclear cells. The molecular diagnostics included genetic variants of *CFH, CFHR1, CFHR2, CFHR3, CFHR4, CFHR5, C3, CFI, MCP, CFB, THBD, DGKE*, and *CFP*, high-risk *CFH* polymorphisms *[CFH-H3]*, high-risk *MCP* polymorphisms *[MCPggaac]*, and *CFH-CFHR* rearrangements.

### Eculizumab Therapy

Treatment with eculizumab in our hospital was indicated for patients with diagnostic criteria for hrTA-TMA who do not respond to CNI withdrawal and/or treatment of possible triggers (e.g., active viral infection). The initial dosing schedule was based on pediatric recommendations for atypical hemolytic uremic syndrome (aHUS) and subsequently modified according to clinical and laboratory findings (15).

All patients received antibiotic prophylaxis with amoxicillin according to the hospital's guidelines, and those without contraindications were vaccinated against the encapsulated organisms *Neisseria meningitidis, Streptococcus pneumoniae*, and *Haemophilus influenzae* serotype b (16).

Eculizumab withdrawal was assessed in patients with TA-TMA resolution.

### Treatment Response

Response to eculizumab treatment was evaluated by monitoring renal function, BP, proteinuria, thrombocytopenia, anemia, transfusion requirements, presence of schistocytes, and lactate dehydrogenase (LDH).

To assess complement blockade, sC5b-9 and CH50 were measured prior to initiation of eculizumab and at least twice a month during treatment. Levels of sC5b-9 ≤ 303 ng/ml and CH50 ≤ 13 U/mL (the lowest cutoff detected) were used to indicate blockade.

Patients who achieved resolution of hrTA-TMA were reassessed 1 year after eculizumab discontinuation using the same clinical and laboratory parameters.

## Results

Between January 2016 and December 2020, 178 HSCTs (147 allogeneic and 31 autologous) were performed at our hospital. Four pediatric patients diagnosed with hrTA-TMA were included in this study. Their demographic, clinical, and diagnostic characteristics are summarized in Table 2. They all had a negative DAT and ADAMTS-13 activity > 10%.

**Table 2:** Demographic and disease characteristics.

**Variables**	**Patient 1**	**Patient 2**	**Patient 3**	**Patient 4**
Sex	M	F	F	F
Age at HSCT (years)	1	14	12	17
Diagnosis	AML	Severe aplasic anemia	B-cell ALL	B-cell ALL
Type of HSCT	Allogeneic	Allogeneic	Allogeneic	Allogeneic
Type of donor	Haploidentical	Unrelated (9/10)	Haploidentical	Unrelated (9/10)
Stem cell source	BM	BM	BM	BM
HSCT conditioning regimen	RIC	RIC	MA	MA
Total body irradiation	No	Yes	Yes	Yes
Interval between HSCT and hrTA-TMA diagnosis (days)	307	136	158	97
Immunosuppressive therapy at hrTA-TMA diagnosis	MMF	MMF, GC	Cyclosporine, GC	GC
Renal replacement therapy at hrTA-TMA diagnosis	–	–	–	–
Active GVHD at hrTA-TMA diagnosis	–	Cutaneous grade II	Cutaneous and gastrointestinal grade II/III	–
Active viral/fungal infections at hrTA-TMA diagnosis	–	Polyomavirus BK y RSV	–	CMV
Active antiviral treatments at hrTA-TMA diagnosis	–	Acyclovir^†^	Acyclovir^†^	Ganciclovir
**Diagnostic criteria for hrTA-TMA**				
Affected organs	Kidney	Kidney and lung	Kidney	Kidney
Confirmation of TMA by renal biopsy	√	–	√	√
LDH elevated above upper limit for age	√	√	√	√
Proteinuria(urine protein/urine creatinine ratio > 0.2 mg/mg)	√	√	√	√
Nephrotic range proteinuria(urine protein/urine creatinine ratio ≥ 2 mg/mg)	√	√	√	–
Hypertension	√	√	√	√
Number of antihypertensive drugs	2	3	3	4
*De novo* thrombocytopenia	–	√	√	√
*De novo* anemia	√	√	√	√
Increase in transfusion requirements	√	√	√	√
Presence of schistocytes in peripheral blood	–	√	√	–
Elevated plasma concentration of sC5b-9 (> 303 ng/ml)	√	√	√	√
**Complement profile**				
Biochemical and immunological profile	Normal	Unrealized	Normal	Pending results
Genetic profile	Carrier of risk haplotype in CFH-H3	Unrealized	Normal	Pending results
**hrTA-TMA treatments before eculizumab**				
Withdrawal of calcineurin inhibitors	√	√	√	√
Plasmapheresis	–	–	–	–
Defibrotide	–	–	–	–
Rituximab	–	–	–	–

*AML, acute myeloid leukemia; B-cell ALL, B-cell acute lymphoblastic leukemia; BM, bone marrow; CFH, factor H; CMV, cytomegalovirus; F, feminine; GC, glucocorticoids; GVHD, graft vs. host disease; hrTA-TMA, high-risk transplant-associated thrombotic microangiopathy; HSCT, hematopoietic stem cell transplantation; LDH, lactate dehydrogenase; M, masculine; MA, myeloablative regimen; MMF, mycophenolate mofetil; RIC, reduced intensity regimen; RSV, respiratory syncytial virus; sC5b-9, soluble terminal complement complex; TMA, thrombotic microangiopathy*.

† *In both cases, acyclovir had a prophylactic indication*.

The median interval between HSCT and hrTA-TMA diagnosis was 147 days (range: 97–307 days). Patient 1 was included after a second haploidentical HSCT due to primary graft failure following a mismatched cord blood transplant from an unrelated donor. All four patients had renal impairment (confirmed by renal biopsy in 3 cases) with proteinuria and hypertension under treatment with at least two antihypertensive drugs (median 3, range: 2–4).

Cardiac involvement was monitored by echocardiography in all cases. Patient 2 was diagnosed with severe pulmonary hypertension (PH) associated with pulmonary TA-TMA. Patients 1 and 3 developed left ventricular hypertrophy secondary to systemic hypertension.

The funduscopic examination was normal in all patients, with no signs of hypertensive retinopathy.

All the patients had a history of risk factors for TA-TMA: chemotherapy (n = 4), TBI (n = 3), HLA antigen incompatibility (n = 4) (haploidentical family donor [(n = 2), 9/10 mismatched unrelated donor (n = 2)], initial immunosuppressive treatment with cyclosporine (n = 4), GVHD (n = 4), viral infections (n = 4), and antiviral treatment (n = 4).

The variables related to eculizumab treatment are shown in Table 3.

**Table 3:** Eculizumab treatment data.

	**Patient 1**	**Patient 2**	**Patient 3**	**Patient 4**
Interval between hrTA-TMA and initiation of eculizumab therapy (days)	48	28	49	100
Duration of treatment (weeks)	65	5.7	52	40.6
Number of eculizumab doses given	34	7	29	26
Withdrawal of eculizumab due to resolution of hrTA-TMA	Yes	-	Yes	Yes
Previous vaccination against encapsulated microorganisms^†^	Yes	No	Yes	No
Antibiotic prophylaxis	Amoxicillin	Amoxicillin	Amoxicillin	Amoxicillin
Infections by encapsulated microorganisms during treatment	None	None	None	None
Adverse effects	None	None	None	None

*hrTA-TMA, transplant—associated thrombotic microangiopathy*.

† *Neisseria meningitidis, Streptococcus pneumoniae, and Haemophilus influenzae serotype b*.

**Figure 1:**
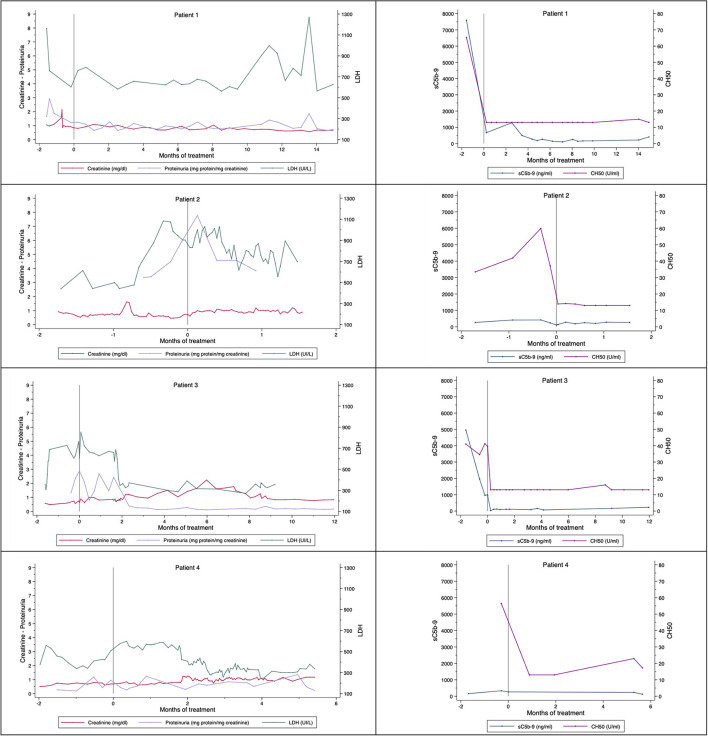
Changes in creatinine, proteinuria, LDH, and markers of eculizumab-induced complement blockade, sC5b-9 and CH50. Month 0 represents the start of treatment with eculizumab. Reference values: sC5b-9 < 303 ng/ml; CH50 < 34 U/ml; LDH 0-248 UI/L. CH50, total complement hemolytic activity; LDH, lactate dehydrogenase; sC5b-9, soluble membrane attack complex.

Figure 1 shows creatinine, proteinuria, LDH, sC5b-9, and CH50 levels before and during eculizumab treatment. Adequate complement blockade (low levels of sC5b-9 and CH50) was achieved after the first dose and during treatment in patients 1, 2, and 3. Patient 4 had normal sC5b-9 levels on initiation of eculizumab, but had high CH50 levels that decreased after the first dose and remained adequate during treatment.

Because we were unable to assess changes in hemoglobin and platelet levels due to the patients' underlying pathology and the need for periodic transfusions, we studied transfusion patterns.

The four cases are presented below in chronological order of TA-TMA diagnosis and initiation of eculizumab therapy.

### Patient 1

At the time of eculizumab withdrawal, patient 1 showed improved renal function (creatinine, 0.69 mg/dL; glomerular filtration rate estimated using the Schwartz formula [eGFR], 60 mL/min) and no longer needed antihypertensive treatment. Proteinuria was 1.65 mg protein/mg creatinine prior to initiation of eculizumab and 0.64 mg protein/mg creatinine after its withdrawal. The patient's anemia had also improved and he no longer needed transfusions, although he was still receiving darbepoetin. His LDH values had also normalized.

The patient continued without signs of active TA-TMA 1 year after eculizumab discontinuation. He had stable creatinine values (creatinine, 0.65–0.69 mg/dl, eGFR 60–65 ml/min), adequate BP control on monotherapy, minimal proteinuria (0.44 mg protein/mg creatinine), and adequate LDH, hemoglobin (without darbepoetin), and platelets, without transfusion requirements.

### Patient 2

Patient 2 was diagnosed with severe, progressive PH at the onset of hrTA-TMA. A diagnostic lung biopsy showed constrictive bronchiolitis and vasculopathy. Although she initially responded clinically to eculizumab and showed improved laboratory values, her clinical status worsened, with progression of PH. She did not respond to eculizumab intensification (weekly administration), and was started on defibrotide and rituximab and sildenafil to treat the PH. Despite aggressive supportive care and therapy with inhaled nitric oxide and vasoactive drugs, she died 76 days after hrTA-TMA diagnosis due to pulmonary edema related to cardiogenic shock.

### Patient 3

On withdrawal of eculizumab, patient 3 showed stable renal function (creatinine, 0.83 mg/dL; eGFR 75 mL/min) and an improvement in BP that enabled withdrawal of two of the three antihypertensive drugs she was taking. She did not have proteinuria (0.16 mg protein/mg creatinine) or schistocytes in peripheral blood. Hemoglobin (on darbepoetin treatment) and platelets were normal and she no longer required transfusions. LDH values had also normalized.

There were no signs of active TA-TMA 1 year after eculizumab withdrawal. Renal function remained stable (creatinine, 0.69 mg/dL; eGFR, > 90 mL/min) and hypertension was controlled with a single antihypertensive drug. The patient continued without proteinuria or schistocytes in peripheral blood and did not require transfusions. Hemoglobin values had increased slightly, although she continued to receive darbepoetin, and platelets were normal. LDH levels remained within the normal range.

### Patient 4

Eculizumab was withdrawn 1 month before the end of the study period in patient 4. This patient had required treatment intensification (administration every 7 or 10 days for 8 weeks) 172 days after initiation due to worsening of renal function, proteinuria, hypertension, and platelet transfusion requirements. Eculizumab levels were monitored during this period of worsening, as the assay was now available at our hospital. The measurements showed adequate levels, confirming adequate dosing.

On withdrawal of eculizumab, the patient's creatinine levels improved (creatinine, 1.1 mg/dL; eGFR, 66 mL/min). Her BP also improved and she was able to stop taking one of four antihypertensive drugs. She had stable proteinuria (0.72 mg protein/mg creatinine). Her hemoglobin and platelet values were adequate and she no longer required transfusions. One month after eculizumab withdrawal, she continued without signs of active TA-TMA.

## Discussion

Our results support the benefits of early complement blockade in patients with hrTA-TMA and add to the little information published on the use of eculizumab in HSCT patients.

All the patients in our study had several risk factors for TA-TMA, confirming the multifactorial nature of this disease (1, 2, 5–8, 11). They had all undergone allogeneic HSCT, which, compared with autologous HSCT, is associated with a higher risk of TA-TMA (2, 6, 9, 10). For one of the patients, this was his second transplant, meaning that he was possibly at increased risk.

Early diagnosis of TA-TMA in the setting of HSCT is challenging and requires experience to differentiate it from other conditions. Organ dysfunction, hypertension, anemia, and thrombocytopenia are all common in this setting and may be caused by drugs (e.g., cyclosporine), infections, or GVHD (2). In addition, schistocytes may not be visible in peripheral blood due to increased vascular permeability (3).

Although TA-TMA is a multisystem disease, the kidneys appear to be the first organ affected (1–3, 12, 13). In our cohort, all the patients had renal involvement and the main manifestations were impaired kidney function, hypertension, and proteinuria. Moreover, recently, Dandoy et al. (9) reported that proteinuria is a high-risk marker TA-TMA. Creatinine may not be an adequate marker of kidney function in pediatric patients due to their low muscle mass. Where possible thus, hrTA-TMA should be diagnosed by renal biopsy, but a risk/benefit assessment should always be performed owing to high risk of bleeding due to thrombocytopenia and hypertension (2, 3, 5).

We did not detect any complement gene mutations in our series, supporting the important role played by non-genetic triggers in the development of TA-TMA in pediatric patients. Nonetheless, it has been demonstrated that secondary TMA is more common in patients with complement mutations (17). Carriage of the heterozygous *CFHR3-CFHR1* deletion, for example, alongside exposure to other TA-TMA risk factors, seems to increase hrTA-TMA susceptibility in allogeneic HSCT recipients (11). In our cohort, just one patient was a carrier of a haplotype associated with an increased risk of TA-TMA. Although this finding does not support the diagnosis of complement-mediated primary, it could facilitate the development of developing TA-TMA when the patient is exposed to risk factors. Moreover, Jodele et al. (11) postulated that genetic screening for complement gene polymorphisms of risk prior to HSCT could be clinically useful for identifying patients with greater susceptibility to hrTA-TMA and for guiding decisions on CNI dose reduction. In the acute setting, involvement of complement in hrTA-TMA by CH50 and/or sC5b-9 elevation is relevant, as eculizumab has been shown to be effective in secondary TMA management (18).

Genetic studies are also important for assessing long-term patient risk, but they cannot support acute management, as it may take months to receive results. One limitation of genetic studies in hrTA-TMA is that not all risk variants are known.

All our patients were hypertensive and required treatment with several antihypertensive drugs (a median number of 3, range: 2–4). We therefore consider that hypertension being treated with two or more drugs in an HSCT patient should alert clinicians to a possible diagnosis of hrTA-TMA.

CNI withdrawal is one of the first steps in a patient with TA-TMA. In our series, cyclosporine was withdrawn in all four patients and replaced with MMF in two. None of the patients responded to this measure, however, and required eculizumab to treat active hrTA-TMA.

As in previous studies, our study shows that eculizumab effectively blocks complement in patients with hrTA-TMA and improves renal and hematological manifestations. The three patients with renal involvement only on initiation of eculizumab (patients 1, 3, and 4) achieved clinical resolution of TA-TMA and normalization of laboratory findings. Patient 2, however, who was diagnosed late with hrTA-TMA and had pulmonary manifestations did not survive.

The median interval between hrTA-TMA diagnosis and initiation of eculizumab therapy was 48.5 days (range: 28–100). It is similar to that reported previously by Jodele et al. (1) (median 33 days, range: 3–122) and de Fontbrune et al. (7) (median 31 days, range: 3–154).

Eculizumab has been shown to be more effective in hrTA-TMA when started early (19). We have described positive outcomes in three patients who were diagnosed early with hrTA-TMA and treated with eculizumab. The other patient, who was diagnosed late and had multisystemic involvement, did not respond. These results support the previous conclusions of previous studies that an early diagnosis and prompt initiation of treatment with eculizumab may improve therapeutic response (1, 7, 9).

Jodele et al. (1, 4) showed that pediatric patients with hrTA-TMA may have higher eculizumab dosage requirements than those with aHUS. Likewise, patients with higher sC5b-9 levels, indicating greater tissue damage and inflammation and a worse prognosis, require intensification of treatment to achieve adequate complement blockade at the beginning of treatment (2, 4). Treatment can be intensified by increasing the dose or reducing the dosing interval. Once sC5b-9 levels have normalized, the intensity can be reduced. Patients 1 and 3 in our series achieved complement blockade and clinical resolution of hrTA-TMA at the recommended dosage for aHUS, despite elevated sC5b-9 values on initiation of eculizumab (sC5b-9, 7593 and 966 ng/ml, respectively). Patient 2 also had elevated sC5b-9 (419 ng/ml) and she required eculizumab intensification due to the severity of her disease at diagnosis. At diagnosis, patient 4 had a slight elevation of sC5b-9 (333 ng/ml). Neverthless, she did not have elevated sC5b-9 on initiation of treatment (263 ng/ml) and she required intensification.

The complement blockade markers proposed to date, sC5b-9 and CH50, appear to be useful for evaluating response to eculizumab, although it should be noted that sC5b-9 levels may be normal. It should also be noted that clinical and laboratory improvements may not be observed for several weeks following adequate complement blockade.

The number of doses of eculizumab given in the responders (34, 29 and 26 doses) is similar to that reported by Schoettler et al. (10) (median 23 doses, range: 3–63), but it is higher than reported by Jodele et al. (13) (median 11 doses, range: 7–20). The duration of eculizumab treatment in the responders (65, 52, and 40.6 weeks) is similar to that reported by Jodele et al. in 2014 (1) (median 47.5 weeks, range: 38–72). Nevertheless, it is longer than reported by Jodele et al. in 2020 (13) (median 66 days, range: 41–110). The shorter duration of treatment in the latest study published by Jodele et al. (13) may be due to the increasing experience in the use of eculizumab in TA-TMA, which could lead to assess an earlier withdrawal of the drug. In our cohort, patient 4, who was the last to start treatment with eculizumab, had shorter treatment duration and received fewer doses.

Eculizumab was withdrawn safely in patients 1 and 3, and favorable laboratory and clinical outcomes were maintained throughout the year of follow-up. The effects of eculizumab withdrawal are not yet assessable in patient 4, but the findings at 1 month following discontinuation are favorable.

As previously reported, eculizumab is well tolerated, and with adequate vaccination and antibiotic prophylaxis, does not increase the risk of infection.

The main limitations and biases of this study are those inherent to any retrospective analysis. Our sample size was small, similar to that most of previous studies, but this is to be expected considering the low incidence of hrTA-TMA in pediatric patients.

The technology to monitor eculizumab levels only became available at our hospital during the treatment of patient 4. While the measurements did not indicate a need for dosage modifications, they did confirm the adequacy of the regimen.

In conclusion, eculizumab appears to be a safe, effective treatment for hrTA-TMA in patients with renal involvement. Early diagnosis and prompt initiation of eculizumab may improve response to treatment. Withdrawal of eculizumab can be contemplated in patients with laboratory findings of adequate complement blockade and clinical TA-TMA resolution.

## Data Availability Statement

The raw data supporting the conclusions of this article will be made available by the authors, without undue reservation.

## Ethics Statement

Ethical review and approval was not required for the study on human participants in accordance with the local legislation and institutional requirements. Written informed consent from the participants' legal guardian/next of kin was not required to participate in this study in accordance with the national legislation and the institutional requirements.

## Author Contributions

LG-G, MB-C, AF-P, and BR-M: data collection. LG-G, MM-L, GA, and CD: bibliographic search. LG-G, MB-C, AF-P, MM-L, BR-M, GA, and CD: data analysis and interpretation. LG-G, MB-C, AF-P, MM-L, and BR-M: writing the manuscript. GA and CD: revision and modification of the manuscript. All authors contributed to the article and approved the submitted version.

## Conflict of Interest

The authors declare that the research was conducted in the absence of any commercial or financial relationships that could be construed as a potential conflict of interest.

## Publisher's Note

All claims expressed in this article are solely those of the authors and do not necessarily represent those of their affiliated organizations, or those of the publisher, the editors and the reviewers. Any product that may be evaluated in this article, or claim that may be made by its manufacturer, is not guaranteed or endorsed by the publisher.

## Abbreviations

AML: acute myeloid leukemia
aHUS: atypical hemolytic uremic syndrome
B-cell ALL: B-cell acute lymphoblastic leukemia
BM: bone marrow
BP: blood pressure
C5b-9: terminal complement complex
CH50: total hemolytic complement activity
CNIs: calcineurin inhibitors
CMV: cytomegalovirus
CFH: factor H
DAT: direct antiglobulin test
eGFR: estimated glomerular filtration rate
F: femenine
GC: glucocorticoids
GVHD: graft vs. host disease
HLA: human leukocyte antigen
hrTA-TMA: high-risk transplant—associated thrombotic microangiopathy
HSCT: hematopoietic stem cell transplantation
LDH: lactate dehydrogenase
M: masculine
MA: myeloablative regimen
MCP or CD46: membrane cofactor protein
MMF: mycophenolate mofetil
MODS: multiorgan dysfunction syndrome
PH: pulmonary hypertension
RIC: reduced intensity regimen
RSV: respiratory syncytial virus
sC5b-9: soluble terminal complement complex
TA-TMA: transplant—associated thrombotic microangiopathy
TBI: total body irradiation
TMA: thrombotic microangiopathy.
